# Intrinsic Rewards for Maintenance, Approach, Avoidance, and Achievement Goal Types

**DOI:** 10.3389/fnbot.2018.00063

**Published:** 2018-10-09

**Authors:** Paresh Dhakan, Kathryn Merrick, Iñaki Rañó, Nazmul Siddique

**Affiliations:** ^1^Intelligent Systems Research Centre, Ulster University, Derry, United Kingdom; ^2^School of Engineering and Information Technology, University of New South Wales, Canberra, ACT, Australia; ^3^Embodied Systems for Robotics and Learning, The Mærsk Mc Kinney Møller Institute, University of Southern Denmark, Odense, Denmark

**Keywords:** intrinsic reward function, goal types, open-ended learning, autonomous goal generation, reinforcement learning

## Abstract

In reinforcement learning, reward is used to guide the learning process. The reward is often designed to be task-dependent, and it may require significant domain knowledge to design a good reward function. This paper proposes general reward functions for maintenance, approach, avoidance, and achievement goal types. These reward functions exploit the inherent property of each type of goal and are thus task-independent. We also propose metrics to measure an agent's performance for learning each type of goal. We evaluate the intrinsic reward functions in a framework that can autonomously generate goals and learn solutions to those goals using a standard reinforcement learning algorithm. We show empirically how the proposed reward functions lead to learning in a mobile robot application. Finally, using the proposed reward functions as building blocks, we demonstrate how compound reward functions, reward functions to generate sequences of tasks, can be created that allow the mobile robot to learn more complex behaviors.

## Introduction

Open-ended learning, still an open research problem in robotics, is envisaged to provide learning autonomy to robots such that they will require minimal human intervention to learn environment specific skills. Several autonomous learning frameworks exist (Bonarini et al., 2006; Baranes and Oudeyer, 2010a,b; Santucci et al., 2010, 2016), most of which have similar key modules that include: (a) a goal generation mechanism that discovers the goals the robot can aim to achieve; and (b) a learning algorithm that enables the robot to generate the skills required to achieve the goals. Many of the autonomous learning frameworks use reinforcement learning (RL) as the learning module (Bonarini et al., 2006; Santucci et al., 2010, 2016). In RL, an agent learns by trial and error. It is not initially instructed which action it should take in a particular state but instead must compute the most favorable action using the reward as feedback on its actions. For many dynamic environments, however, it is not always possible to know upfront which tasks the agent should learn. Hence, sometimes, it is not possible to design the reward function in advance. Open-ended learning aims to build systems that autonomously learn tasks as acquired skills that can later be used to learn user-defined tasks more efficiently (Thrun and Mitchell, 1995; Weng et al., 2001; Baldassarre and Mirolli, 2013). Thus, for an open-ended learning system, autonomous reward function generation is an essential component. This paper contributes to open-ended learning by proposing an approach to reward function generation based on the building blocks of maintenance, achievement, approach and avoidance goals.

Existing literature reveals two common solutions to address the problem of the autonomous reward function design or at least provides a level of autonomy in designing a reward function: (1) Intrinsic motivation (Singh et al., 2005) and (2) reward shaping (Ng et al., 1999; Laud and DeJong, 2002). Intrinsic motivation is a concept borrowed from the field of psychology. It can be used to model reward that can lead to the emergence of task-oriented performance, without making strong assumptions about which specific tasks will be learned prior to the interaction with the environment. Reward shaping, on the other hand, provides a positive or negative bias encouraging the learning process toward certain behaviors. Intrinsic motivation, although promising, has not been validated on large-scale real-world applications, and reward shaping requires a significant amount of domain knowledge thus cannot be considered as an autonomous approach. As an alternative to these solutions, we propose reward functions based on the various types of goals identified in the literature. Although the concept of creating a reward function using goals is not new, this approach is often overlooked and has not been the main focus of the RL community. In our approach, different reward functions are generated based on the type of the goal, and since the reward functions exploit the inherent property of each type of goal, these reward functions are task-independent.

Goals have been the subject of much research within the Beliefs, Desires, Intentions community (Rao and Georgeff, 1995), and the agent community (Regev and Wegmann, 2005). A goal is defined as an objective that a system should achieve (Van Lamsweerde, 2001), put another way, a goal is the state of affairs a plan of action is designed to achieve. Goals range in abstraction from high-level to low-level, cover functional as well as non-functional aspects and can be categorized into hard goals that can be verified in a clear-cut way to soft goals that are difficult to verify (Van Lamsweerde, 2001). Examples of types of goals include achievement, maintenance, avoidance, approach, optimization, test, query, and cease goals (Braubach et al., 2005). Instead of classifying goals based on types, Van Riemsdijk et al. (2008) classify them as declarative or state-based where the goal is to reach specific desired situation and procedural or action-based where the goal is to execute actions. State-based goals are then sub-classified into the query, achieve and maintain goals, and action-based goals are sub-classified into perform goal. RL is already able to solve some problems where some of these kinds of goals are present. For example, well-known benchmark problems such as the cart-pole problem are maintenance goals, while others such as maze navigation are achievement goals. Likewise, problems solved with positive reward have typically approach goal properties, while problems solved from negative reward have avoidance goal properties. The idea of generating reward signals for generic forms of these goals thus seems promising. Based on this logic we propose a domain-independent reward function for each of the goal types. This approach can be applied to the goal irrespective of its origin, i.e., whether the goal is intrinsic, extrinsic or of a social origin. In this paper though, we use the output of an existing goal generation module for a mobile robot (Merrick et al., 2016) to validate the proposed reward functions. We show how the intrinsic reward functions bridge the gap between goal generation and learning by providing a task-independent reward. We further demonstrate how these primitive reward functions based on the goal types can be combined to form compound reward functions that can be used to learn more complex behaviors in agents. Thus, the contributions of this paper are: (1) A proposal for task-independent intrinsic reward functions for maintenance, approach, avoidance and achievement goal types; (2) Metrics for the measurement of the performance of these reward functions with respect to how effectively solutions to them can be learned; and (3) A demonstration of how these primitive reward functions can be combined to motivate learning of more complex behaviors.

The remainder of the paper is organized as follows. In section Background and Related Work, we present a background on the design of reward functions and the solutions for task-independent reward functions found in the literature. In section Primitive Goal-based Motivated Reward Functions, we detail the proposed reward functions based on the goal types, and the metrics we use to measure the agents' performance using those reward functions. In section Experiments for Maintenance, Approach, Avoidance and Achievement Goal Types, we detail experiments to examine the performance of reward functions for maintenance, approach, avoidance, and achievement goal types on a mobile “e-puck” robot. In section Demonstration of how Primitive Goal-based Reward Functions can be Combined, we demonstrate complex behaviors learned from compound reward functions constructed from the autonomously generated primitive functions for each goal type. Finally, in section Conclusion and Future Work, we provide concluding remarks and discuss directions for future work.

## Background and related work

In RL, an agent perceives the state of its environment with its sensors and takes action to change that state. The environment may comprise variables such as the robot's position, velocity, sensor values, etc. These parameters collectively form the state of the agent. With every action that the agent executes in the environment, it moves to a new state. The state of the agent at time *t* can be expressed as:

$$\begin{array}{l} S_{t}=\;\left[s_{t}^{1},\;s_{t}^{2},s_{t}^{3},\ldots ,s_{t}^{n}\right] \end{array}$$

where each attribute $s_{t}^{i}\;$is typically a numerical value describing some internal or external variable of the robot, and *n* is the number of attributes of the state. The agent takes an action *A*_*t*_ to change the state of the environment from the finite set of *m* actions:

$$\begin{array}{l} A=\left\{\;A^{1},\;A^{2},A^{3},{\ldots ,A}^{m}\right\} \end{array}$$

This state change is denoted by event *E*_*t*_, formally denoted as:

$$\begin{array}{l} E_{t}=\left[e_{t}^{1},\;e_{t}^{2},e_{t}^{3},\ldots ,e_{t}^{n}\right] \end{array}$$

where an event attribute $e_{t}^{i}=\;s_{t}^{i}-\;s_{t-1}^{i}$. That is,

$$\begin{array}{l} E_{t}=S_{t}-\;S_{t-1}=\;\left[\Delta (s_{t}^{1}-\;s_{t-1}^{1}),\;\Delta (s_{t}^{2}-\;s_{t-1}^{2}),\ldots ,\Delta (s_{t}^{n}-\;s_{t-1}^{n})\right] \end{array}$$

Thus, an event, which is a vector of difference variables, models the transition between the states. An action can cause a number of different transitions, and an event is used to represent those transitions. Since this representation does not make any task-specific assumption about the values of the event attributes, it can be used to represent the transition in a task-independent manner (Merrick, 2007).

Finally, the experience of the agent includes the states *S*_*t*_ it has encountered, the events *E*_*t*_ that have occurred and the actions *A*_*t*_ that it has performed. Thus, the experience *X* is a trajectory denoted as the following, and it provides the data from which the goals can be constructed.

$$\begin{array}{l} X=\;\left\{S_{0},\;A_{0},\;S_{1},\;E_{1},\;A_{1},\;S_{2},\;E_{2},\;A_{2},\;S_{3},\;E_{3},\ldots \right\} \end{array}$$

### Design of reward functions

In RL, the reward is used to direct the learning process. A simple example of a reward function is a pre-defined value assignment for known states or transitions. For example:

(1) $$\begin{array}{l} r(S_{t})=\{\begin{array}{ll} \;1 & \text{if a paricular state }S_{t}\text{ is reached}\\ \;0 & \text{otherwise} \end{array} \end{array}$$

A more specific, task-dependent example can be seen from the canonical cart-pole domain in which a pole is attached to a cart that moves along a frictionless track. The aim of the agent is to maintain the pole balanced on the cart by moving the cart to the right or left. The reward, in this case, depends on the attributes specific to the task:

(2) $$\begin{array}{l} r(S_{t})=\;-c2^{*}{\left(G^{1}-s_{t}^{1}\right)}^{2}-c3^{*}{\left(G^{2}-s_{t}^{2}\right)}^{2} \end{array}$$

where $s_{t}^{1}$ is the position of the cart and $s_{t}^{2}$ is the angle of the pole with respect to the cart, *G* (with attributes *G*^1^–desired position and *G*^2^–desired angle) is the goal state, and *c2* and *c3* are constants.

For an even more complex task like ball paddling, where a table-tennis ball is attached to a paddle by an elastic string with the goal to bounce the ball above the paddle, it is quite difficult to design a reward function. Should the agent be rewarded for bouncing the ball a maximum number of times? Should the agent be rewarded for keeping the ball above the paddle? As detailed in Amodei et al. (2016), the agent might find ways to “hack the reward” resulting in unpredictable or unexpected behavior.

For some complex domains, it is only feasible to design “sparse reward signals” which assign non-zero reward in only a small proportion of circumstances. This makes learning difficult as the agent gets very little information about what actions resulted in the correct solution. Proposed alternatives for such environments include “hallucinating” positive rewards (Andrychowicz et al., 2017) or bootstrap with self-supervised learning to build a good world model. Also, imitation learning and inverse RL have shown reward functions can be implicitly defined by human demonstrations, so they do not allow a fully autonomous development of the agent.

“Reward engineering” is another area that has attracted the attention of the RL community, which is concerned with the principles of constructing reward signals that enable efficient learning (Dewey, 2014). Dewey (2014) concluded that as artificial intelligence becomes more general and autonomous, the design of reward mechanisms that result in desired behaviors are becoming more complex. Early artificial intelligence research tended to ignore reward design altogether and focused on the problem of efficient learning of an arbitrary given goal. However, it is now acknowledged that reward design can enable or limit autonomy, and there is a need for reward functions that can motivate more open-ended learning beyond a single, fixed task. The following sections review work that focus in this area.

### Intrinsic motivation

Reward modeled as intrinsic motivation is an example of an engineered reward leading to open-ended learning (Baldassarre and Mirolli, 2013). It may be computed online as a function of experienced states, actions or events and is independent of *a priori* knowledge of task-specific factors that will be present in the environment. The signal may serve to drive acquisition of knowledge or a skill that is not immediately useful but could be useful later on (Singh et al., 2005). This signal may be generated by an agent because a task is inherently “interesting,” leading to further exploration of its environment, manipulation/play or learning of the skill.

Intrinsic motivation can be used to model reward that can lead to the emergence of task-oriented performance, without making strong assumptions about which specific tasks will be learned prior to the interaction with the environment. The motivation signal may be used in addition to a task-specific reward signal, aggregated based on a predefined formula, to achieve more adaptive, and multitask learning. It can also be used in the absence of a task-specific reward signal to reduce the handcrafting and tuning of the task-specific reward thus moving a step closer to creating a true task independent learner (Merrick and Maher, 2009). Oudeyer and Kaplan (2007) proposed the following categories for a computational model of motivation: knowledge-based, and competence-based. In knowledge-based motivation, the motivation signal is based on an internal prediction error between the agent's prediction of what is supposed to happen and what actually happens when the agent executes a particular action. In competence-based motivation, the motivation signal is generated based on the appropriate level of learning challenge. This competency motivation depends on the task or the goal to accomplish. The activity at a correct level of learnability given the agent's current level of mastery of that skill generates maximum motivation signal. Barto et al. (2013) further differentiated between surprise (prediction error) and novelty based motivation. Novelty motivation signal is computed based on the experience of an event that was not experienced before (Neto and Nehmzow, 2004; Nehmzow et al., 2013).

### Intrinsically motivated reinforcement learning

Frameworks that combine intrinsic motivation with RL are capable of autonomous learning, and they are commonly termed intrinsically motivated reinforcement learning frameworks. Singh et al. (2005) and Oudeyer et al. (2007) state that intrinsic motivation is essential to create machines capable of lifelong learning in a task-independent manner as it favors the development of competence and reduces reliance on externally directed goals driving learning. When intrinsic motivation is combined with RL, it creates a mechanism whereby the system designer is no longer required to program a task-specific reward (Singh et al., 2005). An intrinsically motivated reinforcement learning agent can autonomously select a task to learn and interact with the environment to learn the task. It results in the development of an autonomous entity capable of resolving a wide variety of activities, as compared to an agent capable of resolving only a specific activity for which a task-specific reward is provided.

Like in RL, in an intrinsically motivated reinforcement learning framework, the agent senses the states, takes actions and receives an external reward from the environment, however as an additional element, the agent internally generates a motivation signal that forms the basis for its actions. This internal signal is independent of task-specific factors in the environment. Incorporating intrinsic motivation with RL enables agents to select which skills they will learn and to shift their attention to learn different skills as required (Merrick, 2012). Broadly speaking, intrinsically motivated reinforcement learning introduces a meta-learning layer in which a motivation function provides the learning algorithm with a motivation signal to focus the learning (Singh et al., 2005).

### Role of goals to direct the learning

Where early work focused on generating reward directly from environmental stimuli, more recent works have acknowledged the advantages of using the intermediate concept of a goal to motivate complexity and diversity of behavior (Merrick et al., 2016; Santucci et al., 2016). It has been shown by Santucci et al. (2012) that using intrinsic motivation (generated by prediction error) directly for skill acquisition can be problematic and a possible solution to that is to instead generate goals using the intrinsic motivation which in turn can be used to direct the learning. Further, it has been argued by Mirolli and Baldassarre (2013) that a cumulative acquisition of skills requires a hierarchical structure, in which multiple “expert” sub-structures focus on acquiring different skills and a “selector” sub-structure decides which expert to select. The expert substructure can be implemented using knowledge-based intrinsic motivation that decides what to learn (by forming goals), and the selector sub-structure can be implemented using competence-based intrinsic motivation that can be used to decide which skill to focus on. Goal-directed learning is also shown to be a promising direction for learning motor skills. Rolf et al. (2010) show how their system auto-generates goals using inconsistencies during exploration to learn inverse kinematics and that the approach can scale for a high dimension problem.

Recently, using goals to direct the learning has even attracted the attention of the deep learning community. Andrychowicz et al. (2017) have proposed using auto-generated interim goals to make learning possible even when the rewards are sparse. These interim goals are used to train the deep learning network using experience replay. It is shown that the RL agent is able to learn to achieve the end goal even if it has never been observed during the training of the network. Similarly, in a framework proposed by Held et al. (2017), they auto-generate interim tasks/goals at an appropriate level of difficulty. This curriculum of tasks then directs the learning enabling the agent to learn a wide set of skills without any prior knowledge of its environment.

Regardless of whether the goals are intrinsic, extrinsic, of social origin, whether they are created to direct the learning or generated by an autonomous learning framework, the approach of using goal-based reward functions detailed in the next section can be applied to them.

## Primitive goal-based motivated reward functions

The basis of our approach in this paper is a generic view of the function in Equation (1) as follows:

(3) $$\begin{array}{l} r(S_{t})=\;\{\begin{array}{c} \;1\text{ if the goal is reached}\\ \;1-\;\varepsilon \text{ otherwise } \end{array} \end{array}$$

where ε is a non-negative constant. The remainder of this section defines different representations of “goal” in Equation (3) and representation of the meaning of “reached.”

### Reward function for the maintenance goal type

A maintenance goal monitors the environment for some desired world state and motivates the agent to actively try to re-establish that state if the distance between the desired state and the current state goes beyond a set limit. For a maintenance goal, an agent's action selection should consider both triggering conditions as well as the constraining nature of the goal (Hindriks and Van Riemsdijk, 2007). The act of maintaining a goal can be never-ending thus making the process continuous or non-episodic.

Consider *G* is the state that the agent desires to maintain. The state is considered as maintained if the distance between the current state and desired state is sufficiently small. The reward at time step *t* can then be expressed as:

(4) $$\begin{array}{l} r(S_{t})=\;\{\begin{array}{c} \;\sigma \text{ if }d\left(S_{t},\;G\right)<\;\rho \;\\ \varphi \text{ otherwise } \end{array} \end{array}$$

where *d(.)* is a distance function, *S*_*t*_ is the current state, *G* is the desired goal state and ρ is a defined distance threshold. The reward for when the goal is maintained is σ and the reward for other time steps is φ, with φ < σ in order to incentivize the agent to find a shorter path to reach the goal state. σ is generally 0 or a positive number to provide positive reinforcement.

We hypothesize that there are various ways in which an agent's performance can be measured with respect to a maintenance goal. For example, the following metrics *M* evaluate the reward function for the maintenance goal type. Each metric is assumed to be measured over a fixed period *T* of the agent's life.

- **Number of Steps for Which the Goal is Maintained (M1)**. This metric counts the total number of times the agent maintains the state for two or more consecutive steps during a period T. Note that since the process of maintaining a goal is continuous, we do not assume the end of a learning episode at the first occurrence of the goal being maintained. For such non-episodic processes, there may be a reason why the maintained state is lost. Thus, this metric provides the measurement of the agent's ability to learn to regain the maintenance goal.
  $$\begin{array}{l} M_{1}=\underset{t=2...T}{\mathrm{count}(t)}\;such\;that\;r_{t}=1\;and\;r_{t-1}=1\; \end{array}$$
- **Number of Steps the Goal is Accomplished (M**_2_**)**. This metric provides an alternative to *M*_1_ and counts the total number of steps for which the agent receives a positive reward. This metric provides the measurement of the number of time steps the agent managed to maintain the goal. A higher value indicates ease of maintainability of a particular goal.
  $$\begin{array}{l} M_{2}=\underset{t=1...T}{\mathrm{count}(t)}\;such\;that\;r_{t}=1\; \end{array}$$
- **Average Number of Steps of Consecutive Goal Maintenance (M**_3_**)**. This measures the length of time (on average) that positive consecutive positive reward is received. This metric also provides an indication of the ease of maintainability of a particular goal. It is calculated by first calculating how many times *J* a goal was reacquired (that is, how many times *r*_*t*_ = 1 *and r*_*t*−1_ ≠ 1) and dividing *M*_2_ as follows:
  $$\begin{array}{l} M_{3}=\frac{M_{2}}{J} \end{array}$$
- **Longest Period of Goal Maintenance (M**_4_**)**. This metric finds the length of the longest stretch for which the agent was able to maintain the goal. This metric indicates the final ability accomplished by the agent in maintaining the goal. Longer stretches indicate better progress in learning to maintain the desired goal state.
  $$\begin{array}{l} M_{4}=\underset{j=1\ldots J}{\max}\left(length\;of\;maintenance\;period\;j\right) \end{array}$$

### Reward function for the approach goal type

An approach goal represents the agent's act of attempting to get closer to the desired world state. The main difference between an approach and maintenance goal lies in the condition of fulfillment. An approach goal is fulfilled when the agent is getting closer to the desired state whereas a maintenance state is fulfilled when the desired state is maintained and not violated. An approach attempt leads to a behavior that functions to shorten the distance, either physically or psychologically between the agent and the desired outcome (Elliot, 2008).

The reward function for the approach goal can be expressed as:

(5) $$\begin{array}{l} r(S_{t})\;=\{\begin{array}{ll} \;\sigma & \text{if }d\left(S_{t},\;G\right)<d\left(S_{t-1},\;G\right)\text{ and }d\left(S_{t},\;G\right)>\rho \\ \;\varphi & \text{otherwise} \end{array} \end{array}$$

where *d(.)*, the distance function is used to check the approach attempt by comparing the distance between the current state *S*_*t*_ and the desired goal state *G* with the distance between the previous state *S*_*t*−1_ and *G*. The second condition of the equation ensures that the distance is more than the defined distance threshold ρ so that “reached” means an approach attempt and not “approach and achieve”. Same as in Equation (4), the reward for when the goal is not reached is φ with φ < σ in order to incentivize the agent to find a shorter path to the goal state.

The following metrics may thus be used to evaluate this reward function for the approach goal type. Each metric is again assumed to be measured over a fixed period *T* of the agent's life. Since the approach and avoidance functions (detailed in section Reward Function for the Avoidance Goal Type) reward the approach and the avoidance attempts irrespective of the distance between the current and the goal state, the cumulative reward for the agent is very high. In order to get a better sense of the proportion of the reward gained per trial, we use percentage in the following metrics.

- **Number of Steps the Goal is Approached as a Percentage of *T (M***_5_***)***. This metric indicates the approachability of the goal, i.e., how easy is it to approach the goal state?
  $$\begin{array}{l} M_{5}=\frac{M_{2}\times 100}{T} \end{array}$$
- **Number of Approach Attempts as a Percentage of *T (M***_6_***)***. The agent is considered to have made an approach attempt if it receives a positive reward for two or more consecutive steps, i.e., signifying that the agent attempted to approach the goal state.
  $$\begin{array}{l} M_{6}=\frac{M_{1}\times 100}{T}\; \end{array}$$

### Reward function for the avoidance goal type

An avoidance goal type is the opposite of the approach goal type. Avoidance is a behavior where an agent stays away or moves away from an undesirable stimulus, object or event (Elliot, 2008). An avoidance goal is considered fulfilled as long as the agent is away from the state that it wants to avoid, and it increases the distance from the state that it wants to avoid. Considering those definitions, the reward function for avoidance goal has two expressions, one that fulfills the condition of moving away from the goal state and other that fulfills the condition of staying away from the goal state, however, in the applications either of the other expressions can be used on their own.

(6) $$\begin{array}{l} r(S_{t})\;=\{\begin{array}{ll} \;\sigma & \text{if }d\left(S_{t},\;G\right)>d\left(S_{t-1},\;G\right)\text{ and }d\left(S_{t-1},\;G\right)>\rho \\ \;\varphi & \text{otherwise} \end{array}\; \end{array}$$

Similar to Equation (5), there are two conditions in Equation (6). The first condition checks for the avoidance attempt, while the second checks that the distance between the previous state *S*_*t*−1_ and the desired goal state *G* is above the defined distance threshold ρ, i.e., the current state is not *G*. Same as in Equation (4), the reward for when the goal is not avoided is φ with φ < σ.

Both the metrics *M*_5_ and *M*_6_ are applicable to avoidance goals. In addition, it is also possible to measure:

- **Number of Times Goal Not Avoided *(M***_7_***)***. This is a count of a number of times the agent fails to avoid the goal state.
  $$\begin{array}{l} M_{7}=\underset{t=1\ldots T}{count(t)}\;such\;that\;d\left(S_{t},\;G\right)<\rho \end{array}$$

### Reward function for the achievement goal type

An achievement goal is a state of the world that the agent strives to fulfill (Duff et al., 2006), i.e., the state that the agent wants to bring about in the future. When this target state is reached, the goal is considered as succeeded. The learning process can be restarted with a same/different initial starting state making the process episodic if required.

Similar to Merrick et al. (2016), we use the concept of an event detailed in section Background and Related Work to represent an achievement task. An event (given as *E*_*t*_ = *S*_*t*_ −*S*_*t*−1_) allows the agent to represent a change in its environment. An achievement goal defines changes in the event attributes $e_{t}^{i}\;$ that the agent should bring about. Thus, the reward for the achievement goal can be generated in response to the experience of event *E*_*t*_ as:

(7) $$\begin{array}{l} r(S_{t},S_{t-1})\;=\;\{\begin{array}{c} \;\sigma \text{ if }d\left(E_{t},\;G\right)<\;\rho \;\\ \varphi \text{ otherwise } \end{array} \end{array}$$

where similar to Equations (4–6), ρ is the distance threshold, σ is generally 0 or a positive number to provide positive reinforcement and φ < σ in order to incentivize the agent to find a shorter path to reach the goal state. The metric *M*_2_ is most useful for measuring the performance of this goal type.

The next section uses the metrics proposed in this section to evaluate the goal-based reward functions detailed by Equations (4)–(7).

## Experiments for maintenance, approach, avoidance, and achievement goal types

**Figure 1:**
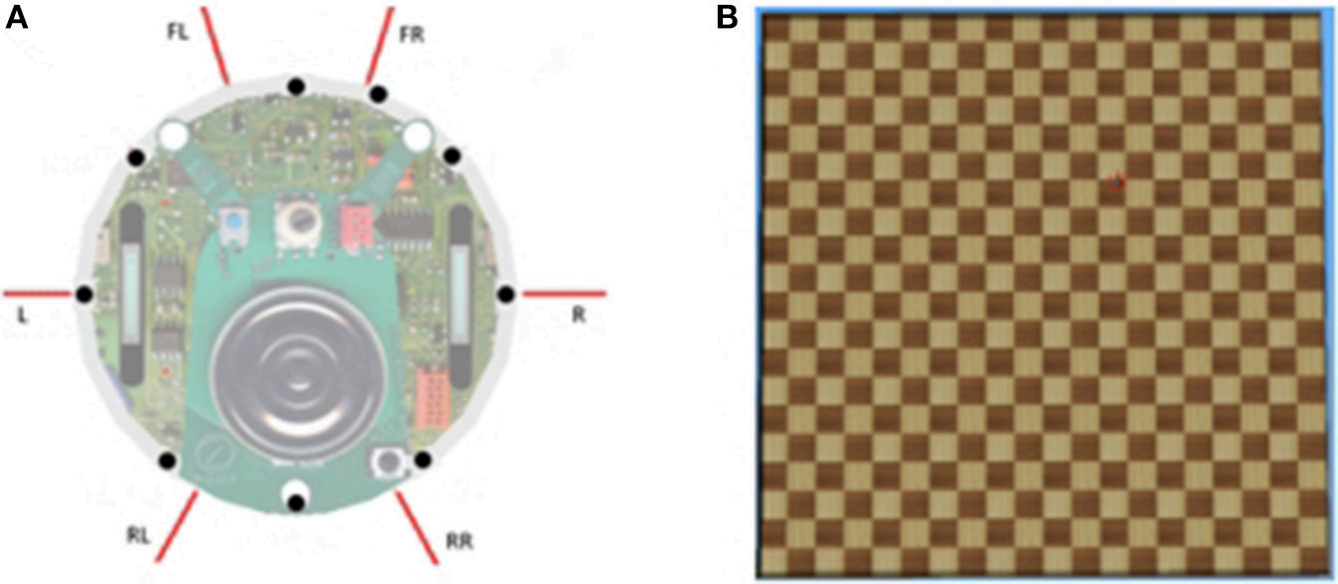
**(A)** e-puck proximity sensors (shown by the red directional lines). **(B)** A sample walled arena.

We used Webots software to simulate an e-puck mobile robot. E-puck is a small differential wheeled mobile robot with eight proximity sensors, of which we used 6. The sensors are labeled in a clockwise direction as *Front-Right, Right, Rear-Right, Rear-Left, Left, and Front-Left*. The red lines in Figure 1A show the direction in which the sensors detect an obstacle. A high sensor reading indicates that an object is close to that sensor. Figure 1B shows a 5 × 5 m square flat walled arena that we use for our experimentation with primitive goal-based reward functions.

The arena, the state, and the action space of the robot are the same as detailed by Merrick et al. (2016). The state of the mobile robot comprises nine parameters: left wheel speed, right wheel speed, orientation, left sensor value, right sensor value, front-left sensor value, front-right sensor value, rear-left sensor value, and rear right sensor value, i.e., the state vector is [ω^R^ ω^L^ θ s^L^ s^R^ s^FL^ s^FR^ s^RL^ s^RR^]. ω^R^ and ω^L^ are the rotational velocities of the right and the left wheels. Their range is –π to π radians per second. θ is the orientation angle of the mobile robot. Its value ranges from –π to π. For our experiments, we use binary values for the proximity sensors with 0 indicating that there is no object in the proximity of the sensor, and 1 indicates that the object is near. The rotational velocities and orientation are discretized into nine values making the state space quite large.

The action space comprises five actions: 1–increase the left wheel speed by δ, 2–increase the right wheel speed by δ, 3–decrease the left wheel speed by δ, 4–decrease the right wheel speed by δ, and 5–no change to any of the wheel speeds. A fixed value of π/2 was used as δ.

In this paper, we use the goals generated for the mobile robot based experiment by Merrick et al. (2016). The main concept of the experience based goal generation detailed in Merrick et al. (2016) is that the agent must explore its environment and determine if the experience is novel enough to be termed a potential goal. Goal generation phase is divided into two stages: experience gathering stage and the goal clustering stage. In the experience gathering stage, the mobile robot moves around randomly in its environment. The states experienced by the robot are recorded. These recorded states form an input to the goal clustering stage which uses simplified adaptive resonance theory (SART) network (Baraldi, 1998). SART is a neural network based clustering technique. It is capable of handling a continuous stream of data thus solving the stability-plasticity dilemma. The network layer takes a vector input and identifies its best match in the network. Initially, the network starts with no clusters. As the data is read, its similarity is checked with any existing clusters. If there is close enough match, it is clustered together else a new cluster is created. As the clusters are created, they are connected to the input nodes (i.e., the recorded experience). The number of clusters created will depend on the vigilance parameter of the SART network. Higher vigilance produces many fine-grained clusters whereas a low vigilance parameter produces a coarser level of clusters. The goals generated by this phase form input for the goal learning phase.

In the learning phase, the robot learns the skills to accomplish the goals. For the goal learning, we use an RL algorithm called Dyna-Q. Dyna-Q (Sutton and Barto, 1998) is a combination of Dyna architecture with RL's Q Learning algorithm. With Dyna-Q, the Q-Learning is augmented with model learning, thus combining both model-based and model-free learning. The RL agent improves its Q value function using both the real experiences with its environment and imaginary experiences (also called planning process) generated by the model of the environment. During the planning process, that is typically run several times for every real interaction with the environment; the algorithm randomly selects the samples from the model (continuously updated using the real experiences) and updates the Q value function. This reduces the number of interactions required with the environment which are typically expensive, especially for robotic applications. The model of the environment for our experiments keeps track of the state *s'* that the mobile robot lands in when it takes a particular action *a* in the current state *s*. The model also keeps track of the reward that the robot receives during that transition. The state transitions for our experiments are deterministic in nature, i.e., when the robot takes action *a* in state *s*, it will always land in a state *s'*. The number of iterations for model learning can be varied as required. We set this parameter to 25, i.e., the algorithm will attempt 25 actions for model learning (using imaginary experiences) before attempting one action with the real environment.

### Maintenance goal learning results

Merrick et al. (2016) used SART based clustering to generate two sets of goals, namely, maintenance and achievement goals. Table 1 lists the set of maintenance goals described by the ID, goal attributes and the meaning of the goal as detailed by Merrick et al. (2016). These goals are the actual states experienced by the mobile robot. This same set of goals are used in section Approach Goal Results and Avoidance Goal Results treated as approach and avoidance type, respectively. Table 4 in section Achievement Goals, lists the set of achievement goals generated by Merrick et al. (2016).

**Table 1:** Experiments and results for maintenance goals.

**ID**	**Goal attributes**	**Meaning of the goal**	***M*_1_**	***M*_2_**	***M*_3_**	***M*_4_**	**Is goal valid?**
G^1^	(2.5, 2.5, 1.8, 0, 0, 0, 0, 0, 0)	Move forward at high speed	37 ± 8	493 ± 91	14 ± 4	154 ± 7	Yes
G^2^	(0.4, 0.4, 1.2, 0, 0, 0, 0, 0, 0)	Move forward at low speed	121 ± 25	568 ± 124	4 ± 1	88 ± 0	Yes
G^3^	(−2.4, −2.4, 1.4, 0, 0, 0, 0, 0, 0)	Move backward at high speed	88 ± 8	888 ± 179	10 ± 2	188 ± 9	Yes
G^4^	(−0.4, −0.4, −1.3, 0, 0, 0, 0, 0, 0)	Move backward at low speed	192 ± 28	866 ± 110	4 ± 0	71 ± 0	Yes
G^5^	(0.0, 0.0, −2.8, 0, 1, 0, 0, 0, 0)	Stop for obstacle in front	1 ± 1	3 ± 3	1 ± 0	5 ± 0	Yes
G^6^	(−0.4, −0.4, 2.9, 0, 0, 0, 0, 0, 0)	Move backward at low speed	142 ± 24	601 ± 106	4 ± 0	37 ± 1	Yes
G^7^	(−0.8, −0.8, 1.6, 0, 0, 0, 0, 0, 0)	Move backward at moderate speed	157 ± 26	848 ± 127	5 ± 0	53 ± 2	Yes
G^8^	(0.2, 0.0, 2.4, 1, 0, 0, 0, 0, 1)	Stop for obstacle behind	0 ± 0	0 ± 0	0 ± 0	0 ± 0	Yes
G^9^	(0.0, −0.3, 2.1, 1, 0, 0, 0, 1, 0)	Stop for obstacle at left and back	0 ± 0	0 ± 0	0 ± 0	2 ± 0	Yes
G^10^	(−1.9, −1.9, −2.2, 0, 0, 0, 0, 0, 0)	Move backward at moderate speed	162 ± 23	763 ± 105	4 ± 0	52 ± 2	Yes
G^11^	(0.0, 0.0, 3.0, 0, 1, 1, 0, 0, 0)	Stop for obstacle in front	0 ± 0	0 ± 0	0 ± 0	0 ± 0	No
G^12^	(1.2, 1.2, −2.7, 0, 0, 0, 0, 0, 0)	Move forward at moderate speed	100 ± 18	427 ± 85	4 ± 0	36 ± 1	Yes

Table 1 also shows the results of the experiments for these goals treated as maintenance goals. The columns *M*_1_, *M*_2_, *M*_3_, and *M*_4_ are the metrics detailed in section Reward Function for the Maintenance Goal Type. The goals are states experienced by the mobile robot treated as maintenance goal for these experiments, i.e., the aim of the robot is to maintain these goal states. The e-puck mobile robot simulation was run for 10 trials each of 25,000 steps for each of the 12 goals. Results were averaged over 10 trials, and the standard deviation is also shown in the table. Values of the parameters of Equation (4) were as follows: ρ was 0.9, σ was 1, φ was −1 and *d* was the Euclidian distance. The RL exploration parameter epsilon was set to 0.15, and the decay schedule was linear. When a trial ended, the end position and orientation of the e-puck mobile robot became the start position and orientation for the next trial. However, the RL Q table was reset after each trial, so no learning was carried forward between the trials.

Once the robot reaches the goal state, it maintains it until it comes across adverse conditions, i.e., for *G*^1^ (move forward at high speed), once the goal state is reached, the robot will maintain that state while it is in the open space. However, once it reaches a wall, it is not able to maintain the state. We consider that the robot has learnt to attain the goal if the robot is able to reach the goal state over and over again and remain in that state for two time-steps or more. This is indicated by the column for *M*_1_. This measure is high for *G*^1^, *G*^2^, *G*^3^, *G*^4^, *G*^6^, *G*^7^, *G*^10^, and *G*^12^ indicating that the robot is able to maintain those goals. However, that measure is very low for goal *G*^5^ and zero for *G*^8^ which shows that the robot is not able to learn to maintain those goal states. This is due to the lack of opportunity, i.e., the robot has to be in a specific situation to be able to learn to maintain those goals. Those goals require the robot to be close to a wall, the likelihood of which is small because of the size of the arena.

The measure *M*_1_ for goal *G*^9^, which is a valid goal, is zero. The mobile robot was not able to achieve that goal because of the lack of opportunity. The required situation to learn that goal would be that the robot should find itself in the bottom left corner at a particular orientation. The measure *M*_1_ is zero for *G*^11^ as well. The reason for that is because goal *G*^11^ is an unreasonable goal. According to that state, the wall is close to the Right and Front Left sensors but not Front Right. It is hard to imagine a position of the mobile robot that represents such state. The goals created by SART are the cluster centers. It appears that this is an example of the clustering algorithm creating a hybrid, unreasonable goal which could be either because the granularity of the clusters is coarser than it should be, resulting in the cluster centroid not being a correct representative of the cluster or that invalid states experienced by the robot due to noise. The column “Is Goal Valid?” is marked “No” in this case.

**Figure 2:**
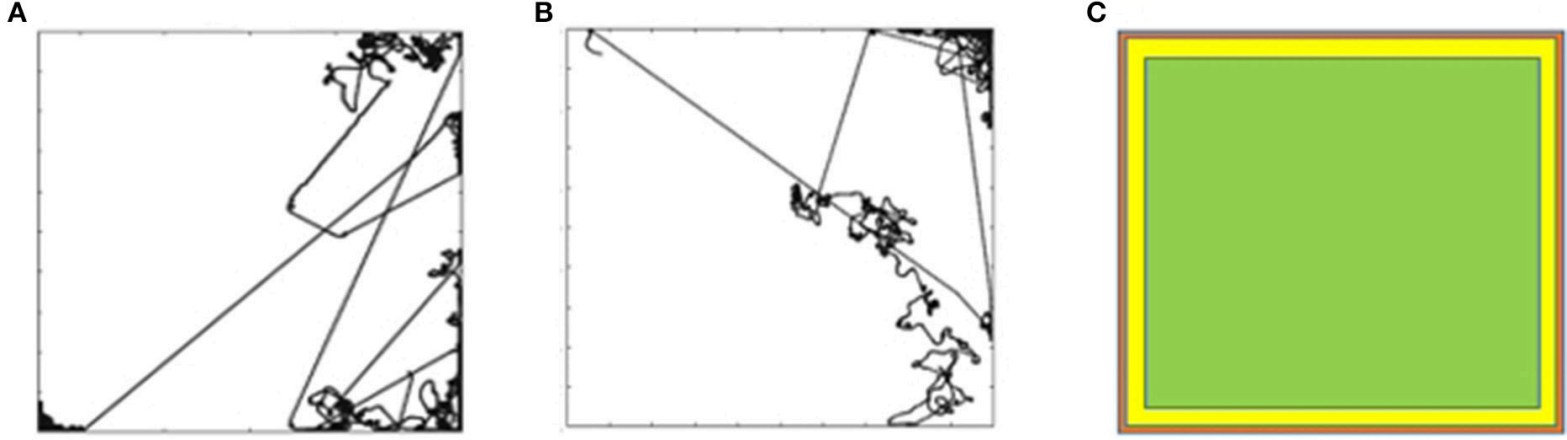
**(A)** Mobile robot trajectory for G1. **(B)** Mobile robot trajectory for G3. **(C)** Likelihood of the reward for G1, G3, and G12.

**Figure 3:**
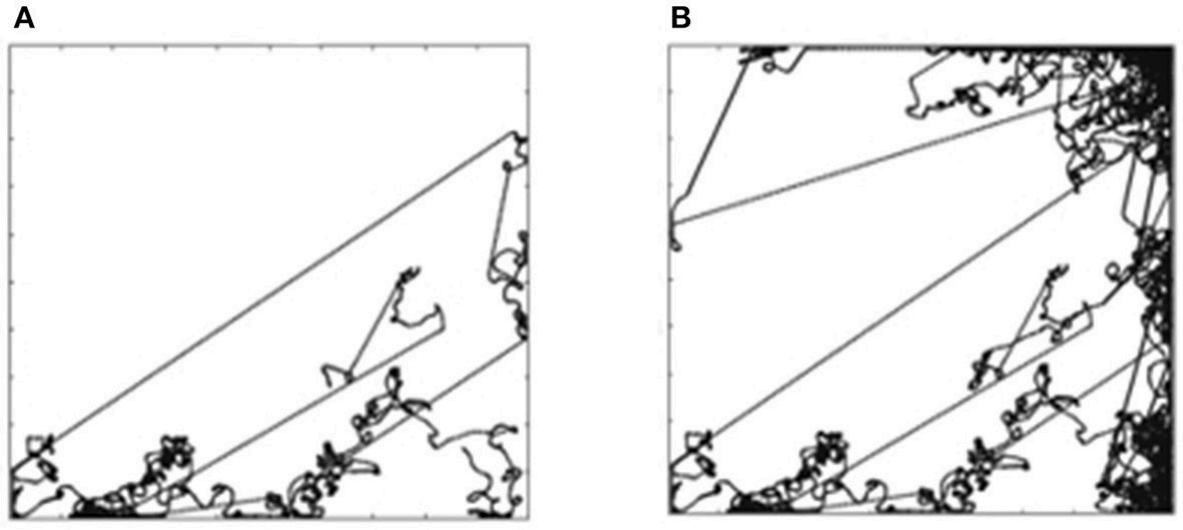
**(A)** Trajectory for G12. **(B)** Simultation for a G12 run for 100,000 steps.

Figure 2A shows a sample trajectory of the mobile robot for *G*^1^. The trajectory is a two-dimensional plot of the path followed by the mobile robot in the arena during the trial. The goal is attained by maintaining a high speed at a particular orientation. The robot receives a positive reward for the time steps that it maintains the goal. It is only possible for the robot to attain *G*^1^ when it is in the open area of the arena. When it reaches the wall, it is no longer able to maintain goal *G*^1^. The robot has to learn to turn around and attain the goal again. This is evident in Figure 2A that shows multiple straight stretches where the robot attains *G*^1^, reaches the wall, tries to turn around and attains the goal again. Figure 2B shows the trajectory of the mobile robot for *G*^3^ (move backward at high speed) and Figure 3A shows the trajectory for goal *G*^12^ (move forward at moderate speed).

For goals *G*^1^, *G*^3^, and *G*^12^ the robot is only able to attain the goals when it is in the open area of the arena. Figure 2C shows the likelihood diagram with the wall shown in orange. In the open area of the arena shown in green, the robot is more likely to attain the goal, i.e., to receive a positive reward. In the area close to the wall (shown in yellow) the likelihood reduces. The probability of the mobile robot to be in the green zone can be calculated as follows for the environment with the size of the board 5 × 5 m and sensor range of e-puck 0.06 m. If we were to discretize the environment into squares of 0.06 m, then there would be 83 × 83, i.e., 6,889 squares in the grid. Green zone for *G*^1^, *G*^3^, and *G*^12^ will consist of 81 × 81, i.e., 6,561 squares. If we were to randomly select a square in the green zone, the probability would be (81 × 81)/(83 × 83) = 95.23%. The orientation and wheel speeds are divided into nine buckets each. Hence the probability of the robot to be in a particular square with particular wheel speed and orientation will be (81 × 81)/(83 × 83 × 9 × 9 × 9) = 0.13%.

For *G*^12^ we let the simulation for one of the trials continue for 100,000 steps, the trajectory of which is shown in Figure 3B. The straight-line trajectory shows that the robot is maintaining the goal of moving forward at a moderate speed, i.e., it is in the region of opportunity (Figure 2C). When the robot reaches the wall, it experiences states that it may not have experienced in the past. However, it eventually learns to attain the goal of moving forward at a moderate speed.

**Figure 4:**
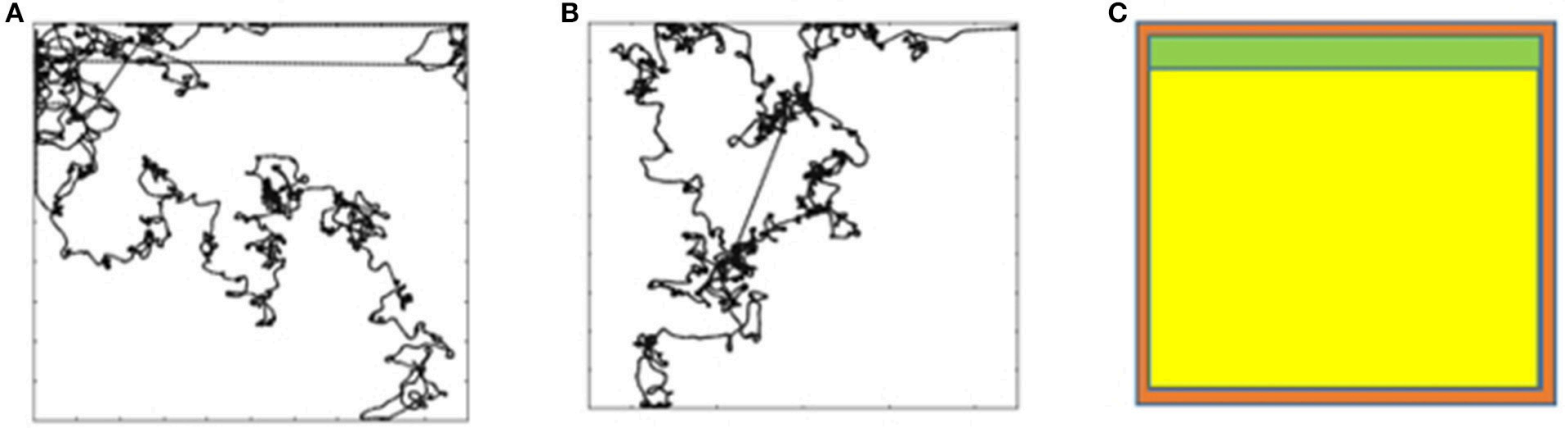
**(A)** Trajectory for G5. **(B)** Simultation for a G8. **(C)** Likelihood of the reward for G5 and G8.

Figures 4A,B shows the trajectory for goal *G*^5^ (stop for an obstacle in front) and *G*^8^ (stop for obstacle behind), respectively. The robot does not learn to attain these goals. The obstacles in the arena are the four walls hence the likelihood of the reward are the areas closer to the wall. Considering the orientation for goals *G*^5^ and *G*^8^, the mobile robot has to be beside the top wall as shown in green in Figure 4C. The probability of the mobile robot to be in a particular square with the orientation required for *G*^5^ or *G*^8^ is (81)/(83 × 83 × 9 × 9 × 9) = 0.002%. This lack of opportunity is the reason why the robot does not learn *G*^5^ and *G*^8^ goals. In order to confirm this hypothesis, we continued the experiments with these two goals with the reduced arena size. The size of the arena was reduced to 0.25 × 0.25 m to increase the opportunity for the mobile robot to be near a wall. In that arena, the probability of the mobile robot to find itself in the required situation is increased by the factor of 400 (20 × 20) to 0.65%, thus increasing its ability to attain *G*^5^ and *G*^8^ goals.

### Approach goal results

Table 2 shows the results of the experiments for the approach goals. The 12 goals and their corresponding goal IDs, goal attributes and the meaning of the goal, are the same as the goals detailed in Table 1. The goals for these set of experiments will be treated as approach goals, i.e., the aim of the robot is to approach those goal states. Values of the parameters of Equation (5) and the method in which experiments were conducted for the approach goals were the same as detailed in section Maintenance Goal Learning Results.

**Table 2:** Experiments and results for approach goals.

**ID**	***M*_5_**	***M*_6_**
G^1^	32.49% ± 0.64	7.56% ± 0.16
G^2^	34.66% ± 0.62	8.00% ± 0.21
G^3^	36.58% ± 0.41	8.39% ± 0.14
G^4^	35.88% ± 0.43	8.52% ± 0.11
G^5^	37.27% ± 0.88	8.84% ± 0.34
G^6^	37.25% ± 0.38	8.74% ± 0.19
G^7^	36.77% ± 0.57	8.76% ± 0.22
G^8^	37.15% ± 0.64	8.73% ± 0.22
G^9^	36.71% ± 0.98	8.60% ± 0.26
G^10^	36.12% ± 0.60	8.24% ± 0.23
G^11^	36.89% ± 0.86	8.74% ± 0.26
G^12^	33.58% ± 0.58	7.40% ± 0.17

The design of the reward function for the approach goal type is such that it rewards an approach attempt. Hence if the agent is getting closer to the goal, it receives a positive reward. Goals, when treated as approach goals, are relatively straightforward to attain as seen in the *M*_5_ column in Table 2 (average number of steps positive reward received as a percentage). In the case of the goal *G*^1^, for instance, the agent receives a positive reward for 32.49% of the time steps. This is because the attempt to approach the goal is rewarded irrespective of the distance between the current state and the goal state. Results also show that all the goals, when treated as approach type, are attainable (even the invalid goals) indicating that it is possible to approach the goal states of each of the 12 goals.

### Avoidance goal results

Table 3 shows the results of the experiments for the avoidance goals. The 12 goals and their corresponding goal IDs, goal attributes, and the meaning of the goal, are the same as the goals detailed in Table 1. The goal states for these experiments are treated as avoidance goals, i.e., the aim of the robot is to avoid those goal states. Values of the parameters of Equation (6) and the method in which experiments were conducted for the avoidance goals were the same as detailed in section Maintenance Goal Learning Results.

**Table 3:** Experiments and results for avoidance goals.

**ID**	***M*_5_**	***M*_6_**	***M*_7_**
G^1^	36.67% ± 0.32	8.63% ± 0.14	45
G^2^	34.88% ± 0.67	8.05% ± 0.25	14
G^3^	32.61% ± 0.41	7.53% ± 0.16	12
G^4^	33.16% ± 0.53	7.62% ± 0.14	12
G^5^	35.60% ± 1.01	8.21% ± 0.30	1
G^6^	34.22% ± 0.94	7.95% ± 0.25	16
G^7^	33.46% ± 0.55	7.75% ± 0.22	13
G^8^	34.90% ± 0.84	8.11% ± 0.18	0
G^9^	35.54% ± 0.64	8.31% ± 0.17	0
G^10^	32.74% ± 0.75	7.52% ± 0.16	6
G^11^	35.46% ± 0.97	8.26% ± 0.33	0
G^12^	37.00% ± 0.77	8.56% ± 0.20	7

The reward function for the avoidance goal type rewards the attempt to avoid the goal, i.e., the agent is moving away from the goal state. As it can be seen in the table, the goals, when treated as avoidance goals, are relatively easy to attain. This is because the attempt to avoid the desired goal state is rewarded irrespective of the distance between the current state and the goal state. Based on the *M*_7_ column (average number of times the goal state was not avoided), it can be said that even the goals that are difficult to attain due to lack of opportunity, when treated as maintenance goals (for example, *G*^5^, *G*^8^, and *G*^9^), are easier to avoid when treated as avoidance goals.

### Achievement goal results

Table 4 lists the set of achievement goals generated by Merrick et al. (2016). The goal ID, goal attributes, and the meaning of the goal are the output of the SART based clustering as detailed by Merrick et al. (2016). The goal state is not the actual state experienced by the mobile robot but is an event as described by $e_{t}^{i}=\;s_{t}^{i}-\;s_{t-1}^{i}$. Thus, for an achievement goal type, the aim of the mobile robot is to learn to achieve the transition described by that event, for example, to learn to achieve goal *aG*^5^ listed in Table 4, which is to increase speed of both wheels, the robot must learn to increase its right wheel speed by 0.9 and left wheel speed by 0.6 in a single transition of state. The goal is considered achieved when the transition $e_{t}^{i}$ is reached regardless of what the state $s_{t-1}^{i}$is.

**Table 4:** Experiments and results for achievement goals.

**ID**	**Goal attributes**	**Meaning of goal**	***M*_2_**	**Is goal valid?**
aG^1^	(0.0, 0.0, 0.0, 0, 0, 0, 0, 0, 0)	Achieve no change	25000 ± 0	Yes
aG^2^	(0.0, 0.0, 0.0, 0, 0, 1, 0, 0, 0)	Detect obstacle in front	43 ± 21	Yes
aG^3^	(−0.1, 0.0, 0.0, 0, 0, −1, 0, 0, 0)	Turn left to avoid obstacle on the right	0 ± 0	Yes
aG^4^	(−0.6, 0.0, −0.1, 0, 0, 0, −1, 0, 0)	Turn left to avoid obstacle on the right	0 ± 0	Yes
aG^5^	(0.9, 0.6, 0.0, 0, 0, 0, 0, 0, 0)	Increase speed of both wheels	6521 ± 268	Yes
aG^6^	(−0.1, 0.1, 0.1, 0, 0, 0, 0, 0, 0)	Turn left	0 ± 0	Yes
aG^7^	(0.1, 0.0, −0.1, 0, 0, 0, 0, 0, 0)	Turn right	0 ± 0	Yes
aG^8^	(0.1, −0.4, 0.0, 0, 0, 0, 0, −1, −1)	Turn right to avoid obstacle behind	54 ± 17	Yes
aG^9^	(−0.3, 0.4, −0.3, 0, 0, −1, −1, 0, 0)	Turn left to avoid obstacle on the right	0 ± 0	Yes
aG^10^	(0.0, 0.5, 0.2, 0, 0, 1, 0, 0, 0)	Turn left to detect obstacle on the right	29 ± 16	Yes
aG^11^	(−0.6, −0.8, −0.2, 0, 0, −1, 0, 0, 0)	Turn right to avoid obstacle	10 ± 4	Yes
aG^12^	(0.0, 0.7, 0.3, 0, −1, 1, 0, 0, 0)	Turn left to sense obstacle on right	0 ± 0	No
aG^13^	(0.2, −0.8, −0.4, 0, 0, 0, 0, 1, 0)	Turn right to sense obstacle on left	12 ± 4	Yes
aG^14^	(0.0, 0.6, 0.1, 0, 0, 0, 0, 1, 1)	Turn to detect obstacle behind	0 ± 0	Yes
aG^15^	(0.0, −0.1, 0.0, 0, 1, 1, 0, 0, 0)	Turn right to sense obstacle in front	0 ± 0	Yes
aG^16^	(1.0, 0.5, 0.1, 0, 1, 0, 0, 0, 0)	Turn right to sense obstacle on left	0 ± 0	Yes
aG^17^	(0.7, 0.9, 0.3, 0.0, −1, 0, 0, 0, 0)	Turn left to sense obstacle on left	18 ± 3	Yes
aG^18^	(1.2, 0.5, −0.1, 0, −1, 0, 0, 0, 0)	Turn to avoid obstacle on left	0 ± 0	No
aG^19^	(0.2, 2.7, −0.2, 0, −1, 0, 0, 0, 0)	Turn to avoid obstacle on left	0 ± 0	No
aG^20^	(−1.7, −0.5, 0.1, 0, 1, 0, 0, 0, 0)	Turn to detect obstacle on right	0 ± 0	No
aG^21^	(−0.7, −1.2, −0.3, 0, 1, 0, 0, 0, 0)	Turn to detect obstacle on left	0 ± 0	Yes
aG^22^	(1.4, 2.0, 0.2, 0, 0, 0, 0, 0, 0)	Turn left	0 ± 0	No

Table 4 also shows the results of the experiments (with a 95% confidence interval) for the achievement goals. The e-puck mobile robot simulation was run for 10 trials for each goal with 25,000 steps in each trial. Parameters of Equation (7) were same as in the above experiments, i.e., ρ was set to 0.9, σ set to 1, φ set to −1 and *d* was the Euclidian distance. Also, the RL exploration parameter epsilon was set to 0.15 with a linear decay schedule. For achievement goals too, when a trial was finished the next trial started at the same position and orientation of the e-puck mobile robot at which the previous trial ended. The Q table, however, was reset after each trial thus there was no learning carried forward between the trials.

While the robot easily achieved goals *aG*^1^ and *aG*^5^, it could either achieve other valid goals only a few times or not able to achieve them at all. Goals *aG*^2^, *aG*^8^, *aG*^10^, *aG*^11^, *aG*^13^, and *aG*^17^ could be achieved only a few times whereas goals *aG*^4^, *aG*^9^, *aG*^14^, *aG*^16^, and *aG*^21^ could not be achieved at all. The reason for that is due to the lack of opportunity. For example, the mobile robot must be near a wall for the event of detecting an obstacle at the front or turning right to avoid an obstacle behind. The argument made in section Maintenance Goal Learning Results regarding reducing the size of the arena to increase the opportunity for learning is valid here too.

Goals *aG*^3^, *aG*^6^, *aG*^7^, and *aG*^15^ could not be achieved due to the granularity of discretization. For the experiments in this paper, the wheel speed and orientation are discretized into nine values ranging from –π to π. The wheel speed difference for the events for those goals was too small hence when discretized; the values returned are 0 resulting in no change to the wheel speed, i.e., the event of the robot turning left, or right is not detected. For example, consider *aG*^7^ where the goal is to turn right by increasing the right wheel speed by 0.1 (also achieving the change in orientation of −0.1). Discretization of the range of 2π radians into 9 buckets gives the granularity of 0.7 radians, thus making the change of 0.1 radians difficult to detect. This, however, does not mean that the goal is invalid. It is a valid goal, just that, for the robot to be able to learn a goal of such precise transition would require experiments to be run with lower granularity values of wheel speed and orientation, which in turn increases the state space and the size of the Q table and drastically increases the time to learn to achieve those goals.

**Figure 5:**
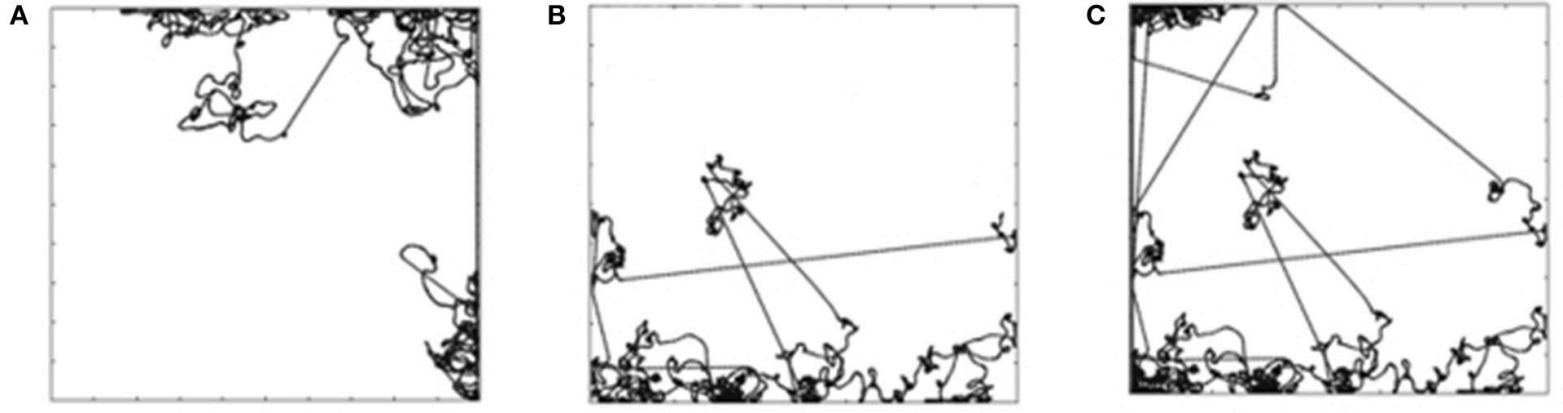
**(A)** Trajectory for aG^5^. **(B)** Trajectory for aG^22^ (run for 25,000 steps). **(C)** Simultation for a aG^22^ run for 100,000 steps.

Figure 5A shows the trajectory for *aG*^5^ (increase speed of both wheels) for one of the trials. The robot learns to attain this goal. In effect, this goal means that the robot has to keep increasing the speed of its wheels. Attaining the maximum speed for both wheels results in the robot not able to achieve the goal anymore and thus receives a negative reward. The robot, however, is again able to attain the goal. This continues until the end of the trial.

Figure 5B shows the trajectory for *aG*^22^ (turn left) for 25,000 steps. The robot is not able to learn to achieve that goal. The trajectory, however, is surprising, showing long stretches of straight line. We let that trial continue for 100,000 steps, the trajectory for which is shown in Figure 5C. The robot still does not learn to achieve the goal. This is because the change in the wheel speed, due to the event (2.0 radians per second for the left wheel speed), is too large for one-time step. In a single step, the maximum change can only be π/2 radians as per the design of the action set. Hence, the goal appears to be unreasonable. The goals *aG*^19^ and *aG*^20^ too appear to be unreasonable for the same reason, and as can be seen from Table 4, they too could not be achieved. *aG*^12^ is unreasonable because goal attributes are showing transition for Right and Front-Left sensors without any transition for Front-Right. It is hard to imagine the location of the mobile robot in the arena that will result in such an event. *aG*^18^ too appears unreasonable because considering the change to the wheel speeds (1.2 and 0.5 radians per second), the transition in the orientation (−0.1 radians) is too small.

Either such unreasonable events experienced by the robot during the experience gathering stage in the experiments run by Merrick et al. (2016) could be due to noise, delay in sensing or that the mobile robot might have got stuck and then unstuck to the wall resulting in an invalid event (*e*_*t*_ = *s*_*t*_ − *s*_*t*−1_) or that the unreasonable events were due to an error in clustering, resulting in cluster centroid not being a correct representative of the cluster. If latter was the case, then it requires reanalysis of the generated clusters. Possible solutions to rectify the incorrect representation of the cluster centroid could be to place a minimum threshold on the cluster size or to shift the cluster centroids to the nearest valid attribute value. In any case, those goals appear unreasonable and are marked as invalid in the table. Based on the findings of the above experiments, for the experiments in the next section, we have removed the orientation attribute from the RL state vector, reduced the size of the arenas and, not used any of the invalid goals.

## Demonstration of how primitive goal-based reward functions can be combined

Not all tasks can be represented as a single goal type. Consider an example detailed in Dastani and Winikoff (2011), if the task for a personal assistant agent that manages a user's calendar is to book a meeting, it can be represented as an achievement goal, however people's schedules change and hence to ensure that the meeting invite remains in the calendar of all the participants, the task is better modeled by a combination of goal types. The goal can be represented as “achieve then maintain” where the aim is to achieve the goal and then maintain it. As another example, consider a wall following mobile robot. The robot has to first approach a wall and then maintain a set distance from the wall either to its left or to its right side. This goal can be represented as “approach then maintain” where the aim of the mobile robot is to first approach the goal state (i.e., a wall to its left or right) and then maintain it. We term this as a compound goal-based reward function, as it can be built from multiple primitive goal-based reward functions.

In this section, we demonstrate compound goal-based reward functions constructed using if-then rules to trigger different primitive reward functions in different states. In this paper, the if-then rules are hand-crafted as we aim to demonstrate that primitive reward functions can be combined to motivate learning of complex behaviors. The question of how to do this autonomously is discussed as an avenue for future work in Section Autonomous Generation of Compound Reward Functions and Conditions for Goal Accomplishment.

### Experimental setup

**Figure 6:**
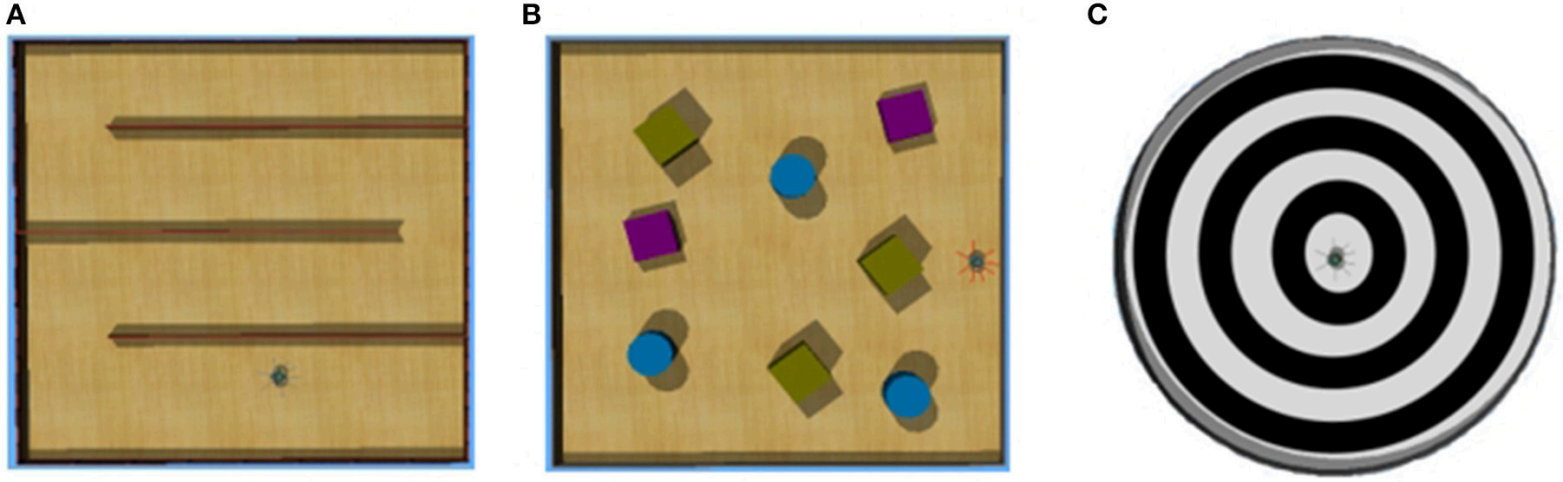
**(A)** Maze arena. **(B)** Arena with obstacles. **(C)** A circular arena with tracks.

To demonstrate compound goal-based reward functions, we use the e-puck robot in three new environments. The environments are as shown in Figures 6A–C. The maze environment, shown in Figure 6A, has walls to form a simple maze. In this environment, the goal of the robot is to follow a wall. That goal is actually a compound goal. In order to achieve the goal, the robot has to learn primitive goals detailed in Tables 1–4. The compound Function 1 details the if-then rules to achieve this goal. The environment with obstacles, shown in Figure 6B, has cylindrical and cuboid objects that act as obstacles. The goal of the robot is to learn to avoid obstacles. The compound Function 2 details the if-then rules to achieve that goal. The third environment is shown in Figure 6C is a circular arena with tracks. The goal of the robot is to learn to follow a track which is detailed by compound Function 3. Experiments were run for the following goals expressed using compound goal-based reward functions. The primitive reward functions shown in the if-then rules (Function 1, Function 2 and Function 3) are the same as in Tables 1–4.

**Function 1:** Wall following goal in the maze arena.

**if** obstacle on the left
aG^17^ – achieve turning left
**elseif** obstacle close on the left
G^1^ – maintain moving forward
**elseif** obstacle on the right
aG^11^ - achieve turning right
**elseif** obstacle close on the right
G^1^ – maintain moving forward
**elseif** obstacle at the front and left /*i.e, corner on the left */
achieve turning right
**elseif** obstacle at the front and right /*i.e, corner on the right */
achieve turning left
**elseif** obstacle at the front
aG^11^ - achieve turning right
**elseif** no obstacle nearby
G^1^ – maintain moving forward
**end**

**Function 2:** Obstacle avoidance goal in the arena with obstacles.

**if** obstacle on the left
aG^13^ – achieve turning right
**elseif** obstacle on the right
aG^4^ - achieve turning left
**elseif** obstacle at the front and/or side
aG^11^ - achieve turning right
**elseif** obstacle at the back
G^1^ – maintain moving forward
**elseif** no obstacle anywhere nearby
G^1^ – maintain moving forward
**end**

**Function 3:** Track following goal in the circular arena with tracks.

**if** the obstacle anywhere nearby
aG^11^ - achieve turning right
**elseif** track to the left
achieve turning left
**elseif** track to the right
achieve turning right
**elseif** on the track
G^1^ – maintain moving forward
**end**

We use the same Dyna-Q algorithm that is detailed in section Experiments for Maintenance, Approach, Avoidance, and Achievement Goal Types. Action selection was using the epsilon-greedy method with epsilon parameter set to 0.1 throughout the learning process. 10 trials were run for each of the experiment with each trial consisting of 25,000 steps.

The state space for this robot is different from that in section Experiments for Maintenance, Approach, Avoidance, and Achievement Goal Types. In addition to the six distance sensors as detailed in the experiments in section Experiments for Maintenance, Approach, Avoidance, and Achievement Goal Types, we also use the ground sensors for these experiments. We label the three ground sensors as *Ground-Left, Ground-Centre, Ground-Right*. The state of the mobile robot comprises of following parameters: left wheel direction, right wheel direction, left sensor value, right sensor value, front-left sensor value, front-right sensor value, rear-left sensor value, rear right sensor value, ground left sensor value, ground center sensor value and ground right sensor value. The state is a vector represented by [ω^R^ ω^L^ s^L^ s^R^ s^FL^ s^FR^ s^RL^ s^RR^ s^GL^ s^GC^ s^GR^]. ω^R^ and ω^L^ are the rotational velocities of the right and the left wheels that are discretized to binary values with 1 indicating that the wheel is moving forward and 0 indicating that it is moving backwards. For the proximity sensors, we use binary values with 0 indicating that there is no object in the proximity of the sensor and 1 indicates that the object is near. For ground sensors as well, we use binary values with 0 indicating that the sensor is detecting light color and 1 indicating that it is indicating dark color.

The action space comprises of three values: 1–turn left, 2–move forward, and 3–turn right.

### Results

Table 5 shows the results of the wall following, obstacle avoidance, and track following goals. Results were averaged over 10 trials, and its standard deviation is shown. The metrics used to measure agent's performance are the same as the ones defined in section Primitive Goal-based Motivated Reward Functions however here the metrics *M*_1_, *M*_2_, *M*_3_ and *M*_4_ measure cumulative reward gained by the agent for all the primitive goals combined, i.e., the measurement for the compound goal-based reward.

**Table 5:** Results for compound goals.

**ID**	**Goal Description**	***M*_1_**	***M*_2_**	***M*_3_**	***M*_4_**
G^1^	Wall following	1373 ± 29	16833 ± 115	10 ± 0	78 ± 6
G^2^	Avoiding obstacles	747 ± 24	13613 ± 109	11 ± 0	81 ± 8
G^3^	Following a track	992 ± 24	14634 ± 127	9 ± 0	74 ± 8

**Figure 7:**
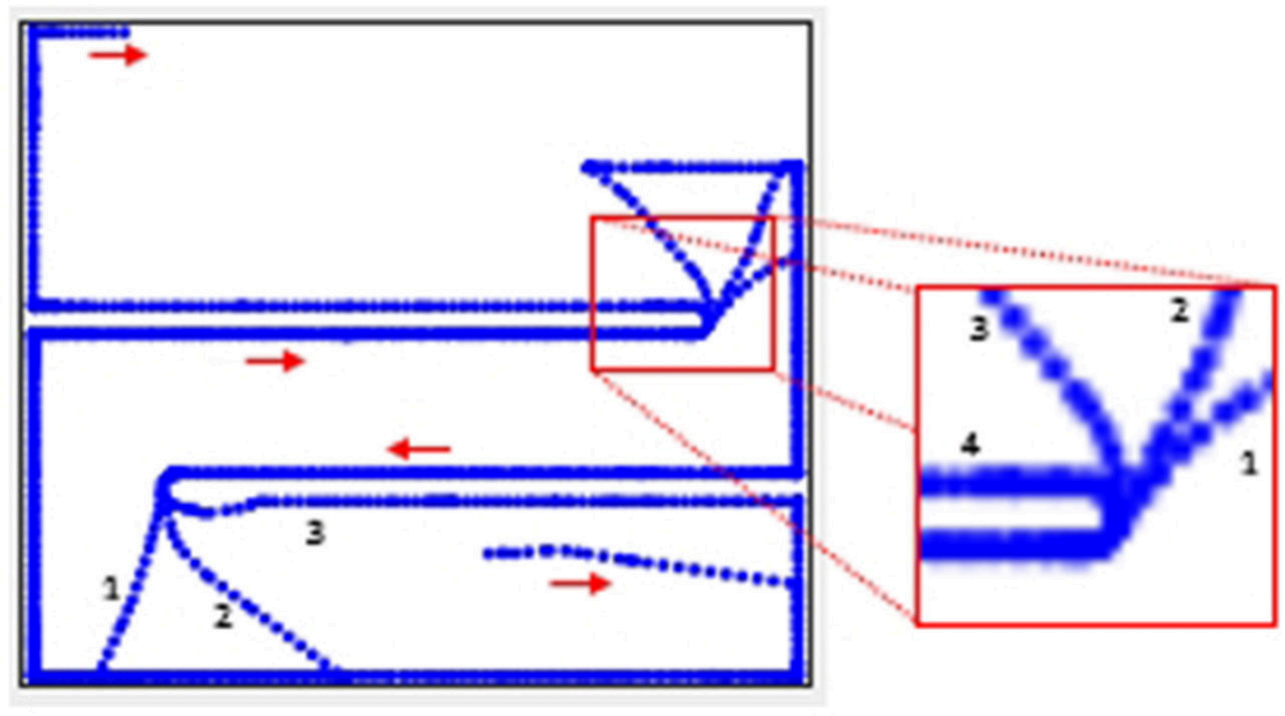
Trajectory for wall following goal in the maze arena.

Figure 7 shows the trajectory for one of the trials of the mobile robot learning to follow the wall using compound goal-based reward function (Function 1). The function comprises of a combination of achievement and maintenance goal types each of which are triggered in a specific situation. When there is no wall in the proximity, the robot is learning to move forward. Once it is near the wall (either to the left or the right), it learns to follow the wall on that side. When it reaches the edge of the wall, it is not able to follow it around for the initial two or three attempts however eventually learns to follow the wall around and continues to follow the wall as shown in the zoomed-in section of Figure 7. Trajectory labeled 4 in the zoomed-in section of Figure 7 is the one where the agent follows the wall all the way around.

**Figure 8:**
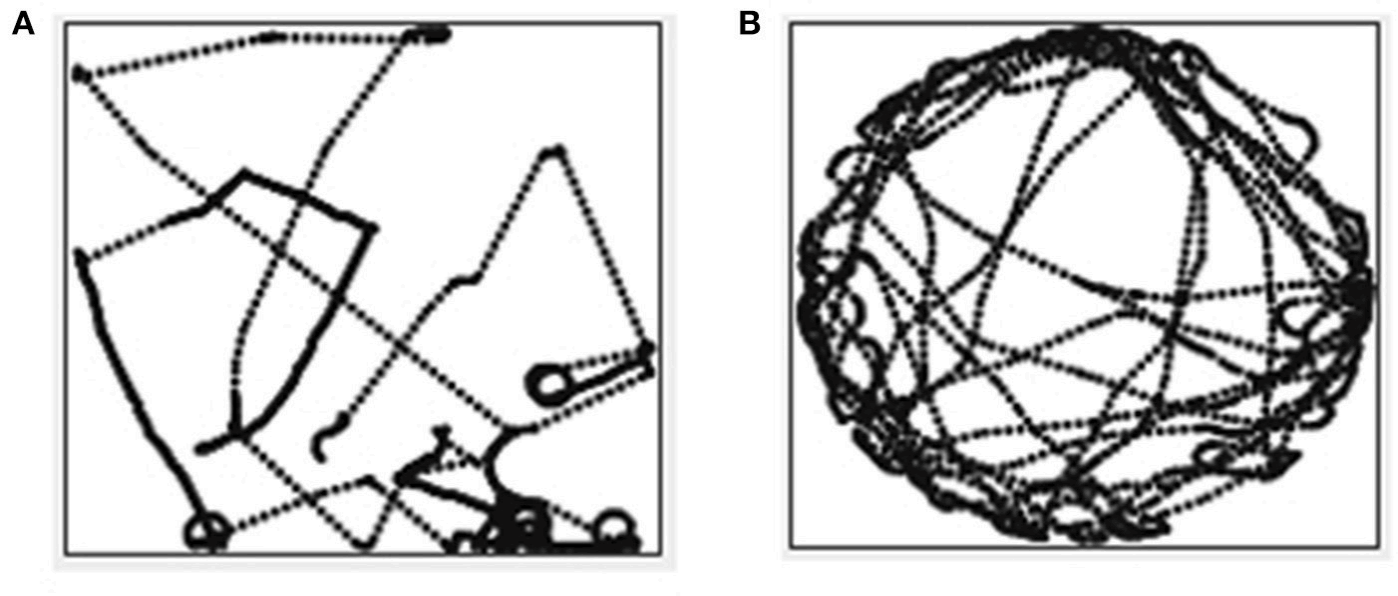
**(A)** Trajectory for obstacle avoidance goal in the arena with obstacles. **(B)** Trajectory for track following goal in the arena with tracks.

Figure 8A shows the trajectory for one of the trials of the mobile robot learning to avoid obstacles using the compound goal-based reward function (Function 2). This function too comprises a combination of achievement and maintenance goal types each of which are triggered in a specific situation. When there is no obstacle nearby, the robot has to learn to move forward. When it is close to an obstacle, it has to learn to turn right and when it has the obstacle at its back it has to learn to move forward, thus moving away from the obstacle. Figure 8B shows the trajectory for one of the trials of the mobile robot learning to follow a track using the compound goal-based reward function (Function 3). When the robot has a wall in its proximity, it has to learn to turn right. When near the track, it has to learn to turn toward the track such that it is entirely on the track. Once on the track, it has to learn to move forward.

## Conclusion and future work

This paper proposed reward functions for reinforcement learning based on the type of goal as categorized by the Belief Desire Intension community. The reward functions for the maintenance, approach, avoidance, and achievement goal types exploit the inherent property of its type, making them task-independent. Using simulated e-puck mobile robot experiments, we show how these intrinsic reward functions bridge the gap between autonomous goal generation and goal learning thus endowing the robot with the capability to learn in an autonomous and open-ended manner.

We present metrics to measure the agent's performance. The measurements show that using the proposed reward functions; all the valid goals will be learnt, some slower than the others due to the lack of opportunity. The goals that are not learnt are either very difficult to learn, unreasonable or invalid. The results also highlight the importance of attributes used in the design of the state vector as it can severely limit the learning opportunity, for example, usage of orientation attribute in the state vector. Although, this paper does not make any claim whether for or against any goal generation techniques, in the future work, the findings from this paper could be used to tune the goal generation technique used by Merrick et al. (2016). We also show that the maintenance goals are easier to learn than the achievement goals. Approach and avoidance goals are even easier due to their inherent nature. This is because, for the maintenance goal, the agent is rewarded only when it can maintain the distance below a certain threshold, whereas, for approach and avoidance goals, the agent is rewarded for the approach or the avoidance attempt irrespective of its distance from the goal.

We further show how rather than treating the goal of a single type, the agent can decide whether it wants to maintain, approach, avoid or achieve the goal based on the situation it is experiencing. This situation specific goal type usage means the agent now knows what it has to learn in a specific situation thus directing the learning. A compound goal-based reward function can be designed by chaining any number of primitive reward functions. This raises following directions for future work.

### Autonomous generation of compound reward functions

This paper demonstrated that primitive goal-based reward functions could be combined using if-then rules to create learnable compound reward functions. However, this raises a question whether it is possible for an agent to self-generate such rules or some other means of combining the primitive reward functions. One potential solution could be for the agent to autonomously determine the structure or regions in its state space each of which relates to a primitive goal. Merrick et al. (2016) have shown how the history of experienced states can be used to generate the goals. In a similar fashion, a coarse level clustering can be run on the experienced states to form these regions in the state space. Once those regions are known, one can then map the regions (primitive goal) with the goal state (compound goal) to enable the generation of the if-then rules. A formal framework is required for identifying complementary or conflicting goals so that complementary goals can be formed into compound reward functions and conflicting goals avoided.

### Conditions for goal accomplishment

We also saw in this work that the agents learn solutions to some goals more effectively when they are in certain situations where the conditions support learning of that particular goal. This suggests that there is a role for concepts such as opportunistic learning (Graham et al., 2012) to maximize the efficiency of learning such that the agent only attempts goals that are feasible in a given situation.

## Author contributions

PD and KM conceived of the presented concept and planned the experiments. PD carried out the experiments under the supervision of KM and IR. PD wrote the manuscript in consultation with KM, IR, and NS. All authors discussed the results, provided critical feedback and contributed to the final version of the manuscript.

### Conflict of interest statement

PD, KM, IR, and NS declare that the research was conducted in the absence of any commercial or financial relationships that could be construed as a potential conflict of interest.
